# Impact of blood perilipin A levels on obesity and metabolic health

**DOI:** 10.1186/s13104-022-06261-3

**Published:** 2022-12-12

**Authors:** Emmanuel K. Ofori, Bright Selorm Letsu, Seth K. Amponsah, John Ahenkorah, Sandra Crabbe, Genevieve Kwao-Zigah, Sylvester Y. Oppong, Patrick Diaba-Nuhoho, Seth D. Amanquah

**Affiliations:** 1 Department of Chemical Pathology, University of Ghana Medical School, Accra, Ghana; 2 Department of Medical Pharmacology, University of Ghana Medical School, Accra, Ghana; 3 Department of Anatomy, University of Ghana Medical School, Accra, Ghana; 4 Korle-Bu Teaching Hospital, Accra, Ghana; 5 International Maritime Hospital, Tema, Ghana; 6 Department of Medicine III, University of Technology, Dresden, Germany

**Keywords:** Perilipin A, Obesity, Insulin resistance, Lipids

## Abstract

**Objective:**

Perilipin A is a common protein that coats lipid surfaces preventing them from being exposed to oxidative damage. Researchers have found little consistency in the relationship between perilipin A levels in the blood and body fat. This study was a cross-sectional observational that looked at circulating perilipin A levels and how they relate to metabolic health.

**Results:**

The participants in this study were 86 individuals with a mean age of 45.5 ± 1.2 years. Multiple clinical and metabolic indicators (age, weight, BMI, total body fat mass, triglyceride, and HOMA-IR) were shown to be inversely associated with perilipin A levels (rho = − 0.32, − 0.37, − 0.40, − 0.45, − 0.33 and − 0.29; *p* < 0.05 respectively). Obese persons were almost six times more likely than non-obese individuals to have lower perilipin A levels (odds ratio = 6.22, CI = 2.35–11.50, *p* < 0.001). Our findings underscore the important role of perilipin A proteins in metabolic health.

## Introduction

Obesity, a complex disorder involving an excessive amount of body fat and its related health-associated problems have become prevalent over the world. Reports suggest obesity has put a significant burden on the world's already inadequate supply of healthcare services [1–5]. Obesity is a risk factor for cardiovascular disease [6, 7], stroke [8, 9], diabetes mellitus [10, 11], high blood pressure [12], musculoskeletal disorders and some cancers [13, 14]. Several environmental and lifestyle variables, most notably overeating and inactivity, are key contributors to occurrences of overweight and obesity in the population [13, 15, 16].

The perilipins are a group of proteins (lipid droplets) that coat neutral lipid surfaces of fat and steroidogenic cells, preventing them from being exposed to oxidative damage [17–19]. Perilipin A is the most characterized of the lipid droplet proteins and functions to regulate the storage and release of lipids, which supply most tissues with fuel [20–22]. According to available data, perilipin A has a connection both with obesity and with the metabolism of adipocytes [23–26]. This has resulted in a significant number of research on obesity being directed toward the biology of lipid droplets.

Despite the aforementioned, available studies on the relationship between circulating perilipin A levels and weight gain are not conclusive and have not always produced consistent results [23, 26, 27]. Some studies have found that obese persons have reduced perilipin A expression on their fat cells when compared to non-obese individuals [23, 27–29]. In separate research conducted by Kern and colleagues (2004) in non-diabetic participants, greater perilipin expression was reported in the obese, which was hypothesized to be associated with increased adipocyte size in the obese subjects [26]. Although the findings of these studies appear to be inconsistent, they do confirm the existence of a link between obesity and perilipin A levels. Without a doubt, enormous benefits would accrue if research could uncover the precise relationship and/or role of perilipin A in obesity-related health issues, especially in a limited resource setting. The aim of the current study was to investigate blood perilipin A concentrations and their association with obesity and metabolic health.

## Main text

### Methods

The research design for this study was observational and cross-sectional. A total of 86 (40 non-obese and 46 obese) community-dwelling volunteers were recruited from the Metropolis District of Greater Accra, Ghana. The individuals were chosen based on their responses to a questionnaire that had information on current health state, age, gender, and anthropometric measurements, among others. The questionnaire had been piloted among 10 non-obese subjects.

**Fig. 1:**
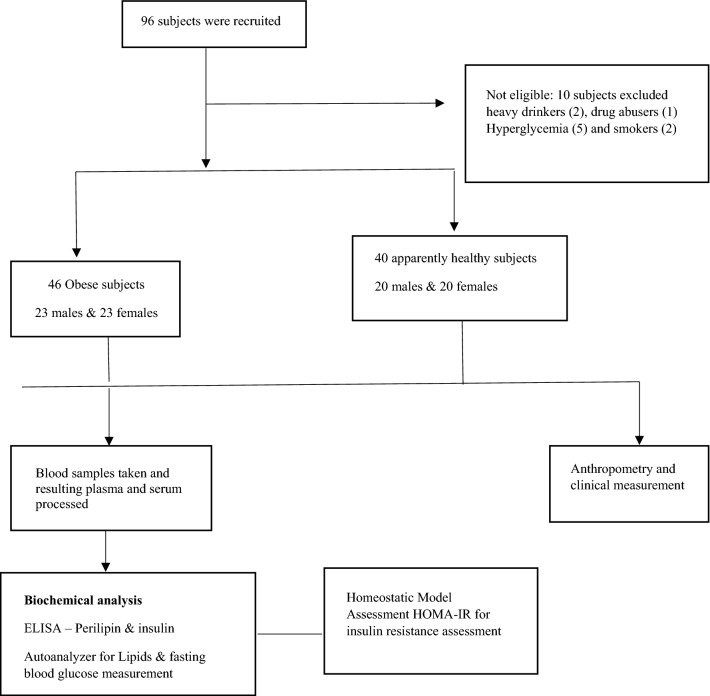
shows participant inclusion and study procedure

Subjects having fasting glucose levels of more than 6.1 mmol/L at baseline, smokers, chronic alcohol users, and those on medication were excluded from the study. Participant inclusion and study procedures are outlined in Fig. 1. All potential subjects had an oral glucose tolerance test (OGTT) conducted to ensure they were not diabetic. Ethical approval for this study was obtained from the Ethical and Protocol Review Committee, College of Health Sciences, University of Ghana (CHS-Et/M.1-P4.7/2016–2017).

The height of participants was measured in centimeters using a wall-mounted stadiometer (Secca, Germany) after subjects had taken off their footwear. The HBF-514 Full Body Sensor Body Composition Monitor and Scale from Omron was used to measure the anthropometrics of the participants in this study (OMRON Healthcare, Netherlands). The monitor needed inputs of age, gender, weight, and height to generate estimations of BMI, percentage of body fat, and visceral fat.

Participants were requested to stand erect and barefooted on the monitor, with their two arms outstretched to grab the handles (electrodes) on either side of the monitor. After stepping on the monitor scale for a few seconds, a tiny electric current of around 5 mA was run through the palms and feet. This is processed to estimate visceral fat and percent body fat by the bioelectric impedance technique. BMI was computed by dividing the weight by the square of the height (Kg/m^2^). A BMI ≥ 30 kg/m^2^ was categorized as obesity [30]. A mercury sphygmomanometer and a stethoscope were used to measure blood pressure after subjects had rested for fifteen minutes.

Venipuncture blood (5 ml) was obtained following an overnight fast (10–12 h). After processing, the sera were stored at − 20 °C until analysis. Levels of perilipin A and insulin in the blood were measured using a solid phase enzyme-linked immunosorbent assay (ELISA) (GenWay Biotech Inc. VA, USA). The test makes use of an enzyme immunoassay of the sandwich kind, which involves the engagement of a double-specific monoclonal antibody. The auto-analyzer for the VITROS system was used to measure fasting blood glucose, total cholesterol, triglycerides, and high-density lipoprotein (HDL) cholesterol levels in the participants (Ortho Clinical Diagnostics, version 5, 1 FS, Rochester, New Jersey, USA). The homeostatic assessment for insulin resistance (HOMA-IR) was computed from fasting glucose and insulin concentrations. An estimated number of 40 persons was adequate to achieve the study objectives at a 5% significant level and this has been described in a prior study [26].

Statistical analysis was carried out using the Statistical Products and Services Solutions (SPSS) version 23 software. Data were reported as mean plus or minus the standard error of the mean (SEM) and at a 95% confidence interval. Spearman's product-moment correlation coefficient (rho) was used to determine the relationship between numeric variables. A multivariate analysis was employed to assess the independent contribution of numerous correlates to the variations in perilipin A levels among obese and non-obese participants. To prevent challenges caused by multicollinearity in multivariate analysis, we used the variance inflation factor (VIF) as previously described [31] to identify a correlation between independent variables and the associated strengths of their correlations. All variables used in the model had VIFs of < 5, which suggests no evidence of potential near multicollinearity. A *p*-value of less than 0.05 was regarded as statistically significant.

## Results

Eighty-six community dwellers took part in this study (45.5 ± 1.2 years, range 30–70 years). Table 1 lists the clinical and biochemical parameters of the study participants. Mean weight and BMI were 88.0 ± 3.2 kg and 32.4 ± 1.1 kg/m^2^ respectively. Independent-samples t-test revealed that BMI, total body fat, visceral fat, HOMA-IR, VLDL and serum levels of triglyceride were significantly higher among the obese participants compared with the non-obese group (*p* < 0.05 for all). In contrast, serum levels of perilipin A and HDL cholesterol were found to be lower in the obese group compared with the non-obese volunteers (*p* < 0.05 for both).

**Table 1:** Clinical and biochemical measurements of study participants

	Obese (n = 46)		Non-obese (n = 40)			Total (n = 86)		
	Mean ± SEM	95% CI		Mean ± SEM	95%CI	Mean ± SEM	95%CI	Range
Age	50.0 ± 1.6	46.9–53.1		40.0 ± 1.7	37.1–43.7	45.5 ± 1.2	43.1.01–48.0	30.0–70.0
Height (cm)	160.4 ± 1.4	157.3–173.2		168.1 ± 1.5	160.8–178.6	164.1 ± 1.2	160.8–175.3	155.7–180.0
Weight (kg)	96.0 ± 1.9*	70.6–152.1		68.2 ± 1.4	51.6–86.3	88.0 ± 3.2	81.6–94.3	50.3–171.2
BMI (kg/m2)	40.47 ± 0.87*	38.72–42.21		22.98 ± 0.41	22.16–23.80	32.4 ± 1.1	30.2–34.5	20.8–52.7
Total Body fat (%)	44.08 ± 1.49*	41.08–47.08		25.50 ± 1.50	22.47–28.53	27.2 ± 2.3	22.5–31.5	5.0–70.0
Visceral fat (%)	18.24 ± 0.86*	16.51–19.97		7.42 ± 1.65	4.08–10.77	13.2 ± 1.1	11.1–15.3	5.0–20.0
Glucose (mmol/L)	5.03 ± 0.07	4.89–5.17		4.97 ± 0.09	4.79–5.15	5.0 ± 0.5	4.8–5.1	3.9–6.1
Total cholesterol (mmol/L)	4.97 ± 0.14	4.68–5.26		4.52 ± 0.12	4.27–4.77	5.42 ± 0.95	4.97–5.96	3.20–8.89
Triglyceride (mmol/L)	1.29 ± 0.07*	1.15–1.44		0.76 ± 0.05	0.67–0.86	1.20 ± 0.49	0.95–1.25	0.34–2.73
HDL-cholesterol (mmol/L)	1.35 ± 0.05*	1.25–1.44		1.65 ± 0.08	1.50–1.80	1.49 ± 0.42	1.40–1.58	0.65–2.84
LDL-cholesterol (mmol/L)	3.03 ± 0.86	2.78–3.29		2.51 ± 0.14	2.24–2.79	2.79 ± 0.96	2.60–2.98	0.79–5.09
VLDL (mmol/L)	0.59 ± 0.03*	0.52–0.67		0.35 ± 0.02	0.31–0.39	0.48 ± 0.24	0.43–0.53	0.16–1.25
Insulin (mlU/ml)	11.84 ± 0.66	10.50–13.17		11.32 ± 0.59	10.10–12.53	11.59 ± 0.37	10.85–12.33	7.23–19.98
HOMA-IR	1.52 ± 0.08*	1.34–1.69		1.437 ± 0.07	1.29–1.58	1.48 ± 0.46	1.40–1.58	0.93–2.47
Perilipin A (pg/ml)	130.8 ± 23.9*	123.7–137.9		159.9 ± 5.2	149.5–170.3	144.3 ± 3.4	97.1–249.0	137.5 –151.1

Data presented as mean ± standard error of the mean (SEM). *BMI* is body mass index, *HDL* is high-density lipoprotein, *LDL* is low-density lipoprotein, *VLDL* is very low-density lipoprotein, and HOMA-IR is the homeostatic model assessment of insulin resistance.

*p < 0.05 significant (unpaired t-test, two-tailed)

Association between measured variables with serum perilipin A and HOMA-IR were determined and are shown in Table 2. Perilipin A levels were negatively associated with several clinical (age, weight, BMI, visceral fat and total body fat, rho = − 0.32, − 0.37, − 0.40, − 0.36, − 0.45; *p* < 0.05 respectively) and metabolic parameters (TCHOL, TG, VLDL and HOMA-IR, rho = − 0.26, − 0.33, − 0.32, − 0.29; *p* < 0.05 for all).

**Table 2:** Association between several correlates with Perilipin A and HOMA-IR

Variables		STUDY SUBJECTS (N = 86)	
		Perilipin A	HOMA-IR
Age	rho	− 0.318*	0.340
	p-value	0.018	0.084
BMI	rho	− 0.398*	0.107
	p-value	0.001	0.229
Weight	rho	− 0.372	0.201
	p-value	0.001	0.353
Visceral Fat	rho	− 0.362	0.108
	p-value	0.001	0.385
Total Body Fat	rho	− 0.448	0.095
	p-value	0.001	0.493
Glucose	rho	− 0.154	− 0.094
	p-value	0.336	0.362
TCHOL	rho	− 0.261	0.130
	p-value	0.020	0.270
TG	rho	− 0.332	0.101
	p-value	0.004	0.405
VLDL	rho	− 0.319	0.100
	p-value	− 0.004	0.409
HDL	rho	0.164	− 0.087
	p-value	0.158	0.540
Insulin	rho	− 0.212	0.915
	p-value	0.062	0.000
Perilipin A	rho	1.000	− 0.287
	p-value	–	0.011

This table shows the association between several correlates with perilipin A and HOMA-IR

*BMI* is body mass index, *TCHOL* is total cholesterol, *HDL* is high-density lipoprotein, *LDL* is low-density lipoprotein, *VLDL* is very low-density lipoprotein, HOMA-IR is homeostatic model assessment of insulin resistance. rho is Spearman’s correlation co-efficient

*p* < 0.05 is significant

Table 3 shows a multivariate analysis of several correlates with perilipin A levels. Obese persons were about six times more likely to have lower perilipin A levels compared with non-obese individuals (Odds ratio; BMI = 6.22, *p* < 0.001, CI = 2.35–11.50).

**Table 3:** Risk assessment of blood perilipin A levels in apparently obese and non-obese subjects

Risk factors	Odds Ratio (Adjusted)	95% CI	*p*-value
Age (yrs.)	0.89	0.31–3.97	0.197
BMI (kg/m^2^)	6.22	2.35–11.50	0.001*
Weight (kg)	1.60	0.18–1.51	0.001
Total Body Fat (%)	1.58	0.18–2.09	0.002
Triglyceride (mmol/L)	0.95	0.11–0.92	0.003
LDL (mmol/L)	0.84	0.13–0.93	0.001
CR (Ratio)	0.82	0.26–1.45	0.160
VLDL (mmol/L)	0.64	0.31–1.75	0.047
HOMA-IR	0.78	0.28–1.82	0.290

This table shows the risk assessment for blood perilipin A levels within the study population

*CI* is confidence interval, *CR* is coronary risk, *LDL* is low density lipoprotein, *VLDL* is very low density lipoprotein, *BMI* is body mass index, Adjusted OR represents the risk of obese participants relative to non-obese participants

p < 0.05 is significant

## Discussion

In this study, the level of perilipin A in blood and its relationship with metabolic health markers in otherwise obese and non-obese persons was investigated. Blood perilipin A levels were shown to be negatively related to age, BMI, percentage of body fat, triglycerides, and insulin resistance (HOMA-IR). This result corroborates other previous findings [26, 28]. In contrast, perilipin 2 (PLIN 2), a protein involved in lipid storage and metabolism in non-adipose tissues correlated positively with BMI, fat mass and insulin resistance in older adults aged 60–80 years [32].

Multivariate analysis in the present study showed that decreased circulating perilipin A concentrations were associated with higher BMI, triglyceride levels, and total body fat. Additionally, risk factors for low perilipin A levels in blood were detected in volunteers with elevated low-density lipoprotein and very low-density lipoprotein cholesterol levels. It has been suggested that obese individuals have lower perilipin expression, indicating that perilipin A may be downregulated in the obese population [23, 33]. Low levels of perilipin A, on the other hand, may have caused an increase in the release of free fatty acids (FFAs), which may have increased the hepatic synthesis of low density and very low-density lipoproteins. There is no apparent explanation for the non-existence of a relationship between perilipin A and visceral fat mass in this study, except for the fact that the participants in this study had good overall metabolic health.

It is generally documented that obesity predisposes people to a wide range of metabolic diseases, including type 2 diabetes [34, 35]. Increased BMI is recognized as a contributing factor in insulin resistance, which ultimately results in the development of type 2 diabetes [30, 36]. Although this association is well established, there is currently considerable skepticism about it because not all obese individuals have insulin resistance [36, 37]. Several studies have suggested that visceral adipose tissue and abdominal subcutaneous adipose tissue are more sensitive indicators of insulin resistance in obese individuals [38, 39]. Obese persons have higher circulating lipids and have more fat deposited in their bodies [40, 41]. High lipid levels in obesity have been related to an increased risk of developing specific diseases such as insulin resistance and atherosclerotic cardiovascular diseases [30, 42]. It has also been shown that obesity is associated with increased levels of inflammation-promoting cytokines such as tumor necrosis factor (TNF-), which is associated with decreased expression of perilipin A in obese people's adipose tissue [43, 44]. The findings of this study demonstrate that blood perilipin A levels are inversely associated with age, body mass index (BMI), percent body fat, triglycerides, and insulin resistance (HOMA-IR). In addition, obesity was an independent risk factor in predicting low blood perilipin A concentrations in adult volunteers. These findings underscore the important role of perilipin proteins in metabolic health.

## Limitations

Some limitations of the current study include the fact that perilipin A levels could not be ascribed to a specific fat depot (for example, subcutaneous or intramuscular fat) since they were measured in the blood. Furthermore, due to the cross-sectional nature of the study, we were unable to establish causality with the associations found. Given the critical function that perilipin proteins play as regulators of lipid metabolism, more studies into the molecular, mechanistic, and synergistic effects they play with other biomarkers are required.

## Data Availability

On reasonable request, the corresponding author will make the datasets that were used and/or analyzed over the course of this investigation available to the interested party.

## Abbreviations

TCHOL: Total cholesterol
HDL: High density lipoprotein
VLDL: Very low-density lipoprotein
LDL: Low density lipoprotein
CR: Coronary risk
CVD: Cardiovascular disease
BMI: Body mass index
SBP: Systolic blood pressure
DBP: Diastolic blood pressure
IR: Insulin resistance
ELISA: Enzyme linked immunosorbent assay
OGTT: Oral glucose tolerance test
